# A discrete event simulation model to evaluate the treatment pathways of patients with cataract in the United Kingdom

**DOI:** 10.1186/s12913-018-3741-2

**Published:** 2018-12-04

**Authors:** Eren Demir, David Southern, Syed Rashid, Reda Lebcir

**Affiliations:** 1 University of Hertfordshire, College Lane, Hatfield, AL10 9AB UK; 2 Pathways Communications, Kennett, CB8 8RW UK; 3 Johnson & Johnson Vision, Wokingham, RG40 3EW UK

**Keywords:** Cataract, Health operations management, Discrete event simulation, National Health Service, United Kingdom

## Abstract

**Background:**

The number of people affected by cataract in the United Kingdom (UK) is growing rapidly due to ageing population. As the only way to treat cataract is through surgery, there is a high demand for this type of surgery and figures indicate that it is the most performed type of surgery in the UK. The National Health Service (NHS), which provides free of charge care in the UK, is under huge financial pressure due to budget austerity in the last decade. As the number of people affected by the disease is expected to grow significantly in coming years, the aim of this study is to evaluate whether the introduction of new processes and medical technologies will enable cataract services to cope with the demand within the NHS funding constraints.

**Methods:**

We developed a Discrete Event Simulation model representing the cataract services pathways at Leicester Royal Infirmary Hospital. The model was inputted with data from national and local sources as well as from a surgery demand forecasting model developed in the study. The model was verified and validated with the participation of the cataract services clinical and management teams.

**Results:**

Four scenarios involving increased number of surgeries per half-day surgery theatre slot were simulated. Results indicate that the total number of surgeries per year could be increased by 40% at no extra cost. However, the rate of improvement decreases for increased number of surgeries per half-day surgery theatre slot due to a higher number of cancelled surgeries. Productivity is expected to improve as the total number of doctors and nurses hours will increase by 5 and 12% respectively. However, non-human resources such as pre-surgery rooms and post-surgery recovery chairs are under-utilized across all scenarios.

**Conclusions:**

Using new processes and medical technologies for cataract surgery is a promising way to deal with the expected higher demand especially as this could be achieved with limited impact on costs. Non-human resources capacity need to be evenly levelled across the surgery pathway to improve their utilisation. The performance of cataract services could be improved by better communication with and proactive management of patients.

**Electronic supplementary material:**

The online version of this article (10.1186/s12913-018-3741-2) contains supplementary material, which is available to authorized users.

## Background

Cataract is an eye disease which occurs when there is a degradation of the optical quality of the crystalline lens affecting the vision ability of individuals [1]. The most important cause of cataract is ageing, but there are other contributing factors including Diabetes, hypertension, obesity, smoking, malnutrition, cumulative exposure to sunlight (ultraviolet-B), and past family history [1–3].

The risk of cataract increases with each decade of life starting at around age 40 and, therefore, it is more prevalent in the ageing population although the disease can affect younger adults if the risk factors are present [3, 4]. As cataract affects vision, it has significant implications for the quality of life of individuals affected by it and their ability to perform normal daily living activities. [5]. If untreated, cataract can lead to complete blindness and according to the World Health Organisation (WHO), the number of people who became blind as a result of cataract was 20 Million in 2010 and it is increasing by 1 Million individuals per year [6].

The situation with regard to cataract in the United Kingdom (UK) is not an exception and future trends are really alarming. In 2010, there were 206,224 and 27,907 people who had partial sight loss or blindness due to cataract, respectively. These figures are expected to rise to 248,504 (partial sight loss) and 32,750 (blindness) by 2020. In addition, the cost impact of dealing with cataract in the UK is stellar. The total cost for treating people with cataract and providing support and social care for those with partial sight loss or blindness was around £950 Million in 2010 and it is expected to reach a total of around £10 Billion over the current decade [7].

There are no medicines to treat cataract and the only option is to undertake a surgical procedure to remove it. The surgery is considered low risk, safe, and cost-effective [2, 3, 8, 9]. Clinical outcomes of the surgery are excellent as the majority of patients reported significantly improved vision and enhanced quality of life following the surgery [1, 10–12].

The number of cataract surgeries in the UK is high and expected to increase in the future. The number jumped from 247,847 in 2001/02 to 336, 967 and 394, 661 in 2011/12 and 2015/16 respectively, making it the highest performed type of surgeries in the UK [6, 7, 13]. This is driven by the ageing population in the UK and the fact that surgery is the only clinical procedure to treat cataract [3, 13]. Future forecasts suggest the number of cataract surgeries will reach 473,994 by 2020 [14]. This trend and its implications for the patients and the country’s health system require a “pause for reflection” from all those involved in the treatment and management of patients with the disease.

The future expected demand for cataract surgery in the UK needs to be appreciated in the context of the country’s healthcare delivery model. Healthcare in the UK is delivered free of charge by the National Health Service (NHS), which is a public entity fully funded from general taxation. However, health budget cuts due to long term “austerity” by successive UK governments in the last decade have left the NHS with serious challenges to deliver care to patients. Putting this against the fact that demand for healthcare in the UK is high (the NHS deal with 1 Million patients every 36 h [15]) and is expected to continue on the same trend and the magnitude of the challenge faced by the NHS becomes clear. If we add to this its massive size and complexity (the NHS employ more than 1.6 Million people [15]), then the need to find innovative and cost effective ways to deliver care is a top priority for the organisation.

In addition to the country level gap between demand and capacity in cataract surgery, financial cuts are forcing NHS trusts to tighten the eligibility criteria for surgery and only patients with serious visual impairment are allowed to have the operation [16]. The extent of the cataract surgery provision crisis is becoming so severe that many NHS ophthalmology clinics, where surgeries are performed, have reached “saturation” levels. Some clinics are running at 100% capacity and others are failing due to financial constraints.

The combined challenges resulting from the upward demand in cataract surgery and the chronic capacity shortage means that a radical change in the way treatment is delivered is needed. Robust and long term cataract service models are required to meet current and future demand. As cataract surgery is considered one of the safest and low risk surgical procedures in developing countries like the UK (the rate of post-surgery complications is around 1% [17]), the challenge stems from the high volume of surgeries to be performed. A new patients’ treatment pathway is required to improve efficiency and increase the number of surgeries taking place at a lower overall cost.

Adopting some aspects of the “lean” operations management approach is a promising way to achieve these objectives. The approach, which has its roots and origins in the manufacturing sector [18], aims to streamline operations and increase capacity by eliminating waste and non-value added activities. In a health management context, this would translate into simplified pathways and increased capacity to provide care to patients [19, 20]. However, despite the attractiveness of lean principles in the severely resource constrained context of the NHS, their adoption has been limited and cataract is not an exception [19]. In one study, [21] explored the efficiency of introducing a lean cataract pathway in a hospital. The results showed a 23% reduction in hospital visits with an increased access to the cataract pathway of 42%. In another study, it was found that the application of lean thinking in hospitals’ cataract services decreased the frequency of hospital visits, lead times, and costs [22].

From an operational perspective, lean principles were applied in a number of ways. Some hospitals implemented a “One stop” service where diagnosis and treatment (cataract surgery) are carried out on the same day of referral [23]. Other hospitals delegated the pre-operative assessment and post-operative assessment to Healthcare Professionals [24]. However, these processes were not sustainable and have been largely abandoned [25]. More recently, cataract care pathways have been altered by introducing new technologies to reduce surgery duration, involving community care units in post-operative assessment, and using phone conversations to perform the pre-surgery assessment [26]. However, it is not clear to what extent these changes have affected the performance of cataract services especially that their adoption has been limited.

A robust evaluation of operational policies and changes to care pathways is, therefore, important. This is very critical in the resource constrained NHS, where there is no luxury of implementing policies without a strong evidence that they lead to improved performance. Discrete Event Simulation (DES) is a modelling technique, which has been applied extensively to this type of health problems to provide evidence of the likely impact of policies prior to their implementation [27–29]. DES represents care pathways structures and resources on a computer software interface and uses the “what-if” scenarios facility to determine the effect of implementing policies on performance. In the context of cataract such scenarios could be, “how many surgeries can be performed per year if we reduce surgery duration by 10%”, and “what is the impact of changes to the utilisation of resources such as theatres (the hospital room where surgeries are performed) and consultation rooms (rooms where doctors and nurses see patients to discuss their medical condition) on total care delivery costs”. DES offers a safe computer based environment to evaluate the implications of such policies before they are implemented, hence avoiding the trap of “doing things and hoping for the best”.

However, despite the rich tradition of DES applications in health, no model has been developed to evaluate the impact of redesigning cataract services. In this context, we developed a DES model for a mid-sized hospital in the UK, which has had significant demand for cataract surgery, extended waiting time for patients, and budget cuts. The aim is to determine if some alternative policies considered by the hospital managers could improve the performance of cataract services from the operational and cost perspectives.

The paper is organised as follows: in the section "Methods", we briefly describe the benefits of DES, and how it differs from conventional modelling approaches. The section also includes the qualitative map portraying the inner workings of the cataract service pathway, model building assumptions and input parameters. All results with model outputs and scenarios are presented in section “Results”. Finally, in sections "Discussion" and “Conclusions”, we discuss the implications of the results, the usefulness of the methodology, and the limitations of the study.

## Methods

Discrete Event Simulation (DES) was selected to represent the pathways related to the management and care of cataract patients. DES is appropriate in this research because of its ability to represent patients at the individual level and tracking their progress along the pathways. DES achieves this by conceptualising patients as entities and their possible states in the pathways as events. As time evolves, patients move between different states (events) and this is captured by the simulation model [30]. In addition, DES enables representation of the inherent uncertainties in the care system (for example the number of patients referred to cataract surgery per week or the chance that a patient will require further surgery due to unexpected post-operative complications). Another distinctive advantage of DES is its ability to allocate attributes to distinguish between individual patients (age, gender, presence of other medical conditions), which can influence their “route” on the care pathway and the treatment outcome.

DES can also represent the mix of resources required for care and treatment, the effect of resources constraints on patients’ progress on the pathways, and the resulting impact on the clinical, operational, and economic performance of the care delivery system. This feature is important in a severely constrained organisation such as the NHS. DES provides also modelling flexibility and can accommodate different managerial rules and policies regarding the allocation of resources including shifts in priorities to deal with bottlenecks or sudden unexpected increase in demand.

However, the “added value” of a DES model lies with its ability to represent alternative pathways configurations and intervention policies. Once a model representing the current situation of the care system is built (baseline model), the DES "What-if" facility can be used to test alternative scenarios reflecting new policies and interventions (for example introducing new technology to reduce cataract surgery process time or shifting 50% of patients from hospitals to local community services for post-surgery assessment). These scenarios can be run on the DES model, which provides accurate information about the expected performance of the care system should the policies and interventions get implemented. This provides policy makers with the required information and evidence regarding the most promising changes to put in place and enhances confidence in the policy making process.

DES has had a long tradition of successful applications in the area of health management spanning over more than 40 years [28, 29, 31]. Specific examples include management of patient flows in hospitals [32], reconfiguration of care delivery services and capacity planning [33–35], management of chronic diseases such as Diabetes and HIV [36, 37], reorganisation of emergency departments [38], cost implications and effectiveness of treatment procedures [39–41], evaluation of screening policies [42], scheduling of treatment procedures [43], and the use of community services in the treatment and management of patients with Parkinson’s disease [44, 45].

### Cataract DES simulation model development

A DES model representing cataract patients’ treatment and management pathways was developed at Leicester Royal Infirmary Hospital, which is one of the biggest and most important hospitals in the UK region of Leicestershire. The model development process included three stages. First, the hospital’s ophthalmology service patients’ data was anonymised and analysed in greater detail. This was followed by the mapping of the hospital’s cataract services structure in collaboration with the management and clinical teams in the service, who provided detailed information about the processes and resources required to manage and treat patients. The findings of the previous two stages were then combined to build the DES model using the SIMUL8 software. To comply with the research university procedures, ethical approval was sought and received from the university research ethics committee before the data collection process started at the hospital.

### Cataract services patients’ pathway

**Fig. 1:**
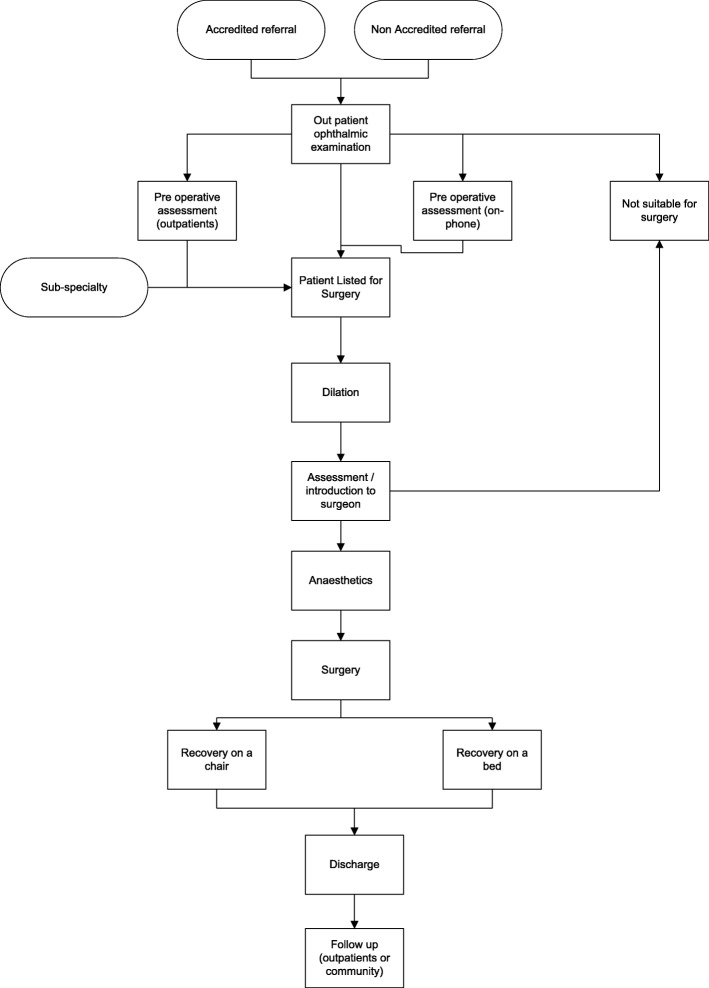
Cataract services patients’ pathways

The objective of the cataract services structure mapping was to determine the steps a patient goes through to receive treatment and care and which resources are required along these steps. This process was informed by a series of semi-structured interviews with cataract service nurses and doctors across a number of clinics. A number of iterations were required to determine the pathways structure and, in every iteration, the latest map of the services was presented to the interviewees, who were asked to comment on the map and determine any gaps and inaccuracies. The map was “frozen” once it was confirmed to be correct by all participants and a diagram of the cataract services patients’ pathway is presented in Fig. 1.

Patients’ journey starts when they are referred to cataract services by an accredited or non-accredited optometrist. Following this, an examination of the eye is carried out by a doctor or a specialised nurse in an outpatient setting, where a number of tests are performed (eg measurement of visual acuity, measurement of intraocular pressure, dilated examination of the cataract). There are four possible outcomes following this examination: 1) patient not suitable for surgery, 2) patient listed for surgery, 3) patient is suitable for surgery but a pre-operative assessment is required, and 4) patient is suitable for surgery and a pre-operative assessment over the phone is required before listing for surgery. Patients could also be listed for surgery if, after visiting another service in the hospital (eg Diabetes services), doctors in these services suspect the presence of cataract. These patients are referred for an examination of the eyes and, if the cataract is confirmed, they are added to the list for surgery.

On the day of surgery, a pre-surgery assessment is carried out by a specialised nurse, which includes dilating and examination of the eye, blood sugar level, blood pressure, temperature, and scanning of the eye and taking the measurements of the lens to be inserted in the eye after the removal of the cataract. Following this assessment and prior to surgery, anaesthesia is performed and this is, in most cases, a local anaesthetic to numb the eye. However, for a small number of patients (eg those with Parkinson’s Disease who cannot stand still during the surgery) general anaesthesia is the preferred option. Surgery is performed by a trained ophthalmologist doctor and lasts for around 20 min. Following surgery, patients who had local anaesthetic are moved to a recovery chair while those who had general anaesthetic are moved to a recovery bed (although a recovery bed is sometimes used for elderly patients even if they had local anaesthetic). The vast majority of patients are discharged on the day of surgery (day-case patients) and a very small number of patients are kept overnight in the hospital for observation. Post-operative follow-up is carried out in the community or in an outpatient setting.

#### Building of the simulation model

The cataract services pathway (Fig. 1) was modelled using the DES software SIMUL8. The software is user friendly and has a graphical interface, which enables representation of the patients, the different treatment and care activities they go through on the pathway, the mix of resources required in every activity, and the allocation rules associated with the management of resources.

**Fig. 2:**
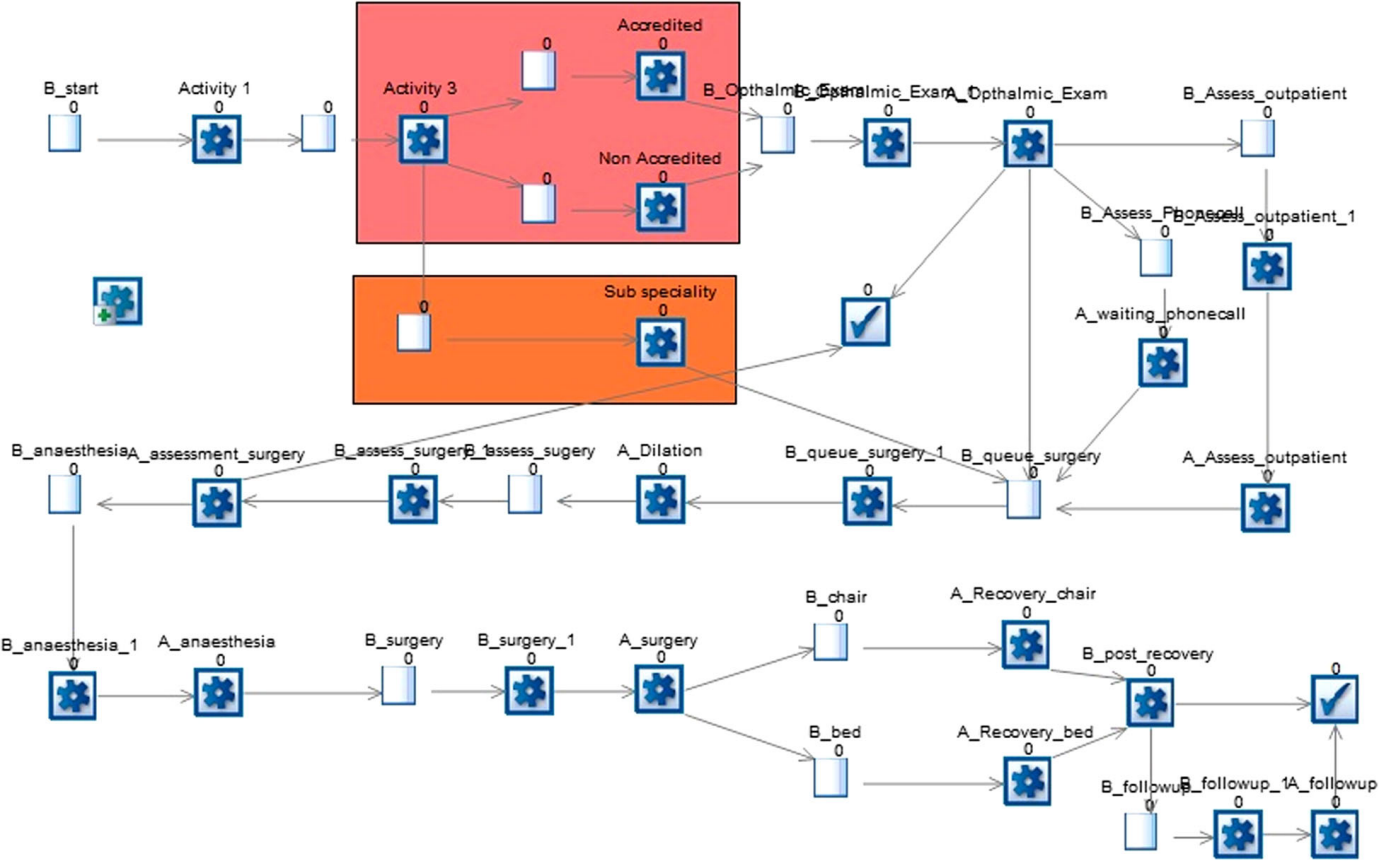
Snapshot of the SIMUL8 DES simulation model

As the purpose of the model is to evaluate the impact of implementing new policies on the operational and cost performance of cataract services, a set of performance indicators relevant to the areas of performance evaluation is included in the model. The indicators are important as they inform the policy making process by providing evidence on the policies expected to lead to the most improved performance. A snapshot of the SIMUL8 model is presented in Fig. 2.

### Data collection and model parameters

Following the building of the simulation model, it was populated with data regarding the values of the model parameters and variables. Two sources were used to determine the model data. The first source is the UK national Hospital Episodes Statistics (HES) dataset, which contains medical and administrative details of all patients admitted to and treated in NHS hospitals in England. UK. This source was useful in determining data regarding the patients and their distribution among the different stages on the care pathway. The analysis of the HES dataset provided also information regarding the probability distributions of pathway related variables such as the waiting time for the eye assessment following referral and waiting time before surgery. The Second source of data for the model is the hospital where the cataract service is located. This source provided data on treatment activities, resources, and the costs of the treatment.

The values of the parameters entered in the model cover all the areas required for the cataract simulation model including demand, diagnosis, treatment, resources, costing, and salary parameters. To determine the level of demand, patient level data over a three year period (2013–2016) was obtained from HES dataset and analysed using statistical software R (library package *forecast*). A series of models were developed to forecast the level of demand (number of patients) for cataract treatment expected over the next 12 months. The forecasted level of demand was then adjusted for population growth rates and collated to estimate future values for 2017 (12 months forecast). To assess the robustness of the forecasting method, the forecasted values for the years 2015 and 2016 were compared to the actual real world values and the forecast accuracy was in the range of 90–99%.

The capacity of cataract surgeries is captured through the parameters “number of lists per theatre per week” and “number of patients per list”. The “number of lists per theatre per week” represents the number of planned sessions (a session is half a day) per theatre per week allocated to cataract surgery as theatres are shared with other services in the hospital. For example, the cataract services may be allocated three “lists” per week (Monday morning, Wednesday afternoon, and Thursday morning). The “number of patients per list” represents the number of cataract surgeries taking place in every list (for example, six surgeries per list). Under this example, the total number of surgeries planned to take place per theatre per week is 18 (3 lists per week each involving 6 patients).

The average hourly cost of staff was derived from two sources: (i) the report published by Personal Social Services Research Unit (PSSRU) known as “Unit Costs of Health & Social Care 2016” [46] and (ii) from the hospital finance office. The revenue generated for each procedure (e.g. cataract surgery, first appointments, follow-ups, etc.) is determined using the UK Department of Health (DoH) “national tariff”. The tariff is the average amount reimbursed by the NHS in England for cataract treatment and set nationally by the UK DoH. The list and values of the model parameters are included in Additional file 1 with this paper.

### Verification and validation of the model

Following completion of the simulation model, verification and validation tests were performed on it to evaluate its robustness before it is used for scenario analysis and evaluation of alternative policies. First, the structure of the model was checked with the hospital cataract services clinical and management teams. A detailed review of the simulation model by these teams identified the aspects of the model, which were not an accurate representation of the real world. The simulation model was updated as a result of the teams’ comments until they were satisfied that it reflected the reality with a high level of accuracy.

The second phase of the model verification and validation process focused on checking the model’s ability to replicate real world observed data. This was carried out by comparing the average total time (in weeks) of patients from initial referral to post surgery discharge. The differences between the real world observations and the simulation model results were within the confidence interval range of 95%. This deemed the model validated and, therefore, suitable to use to evaluate alternative policy scenarios.

## Results

The primary aim of this research is to determine if there are possible changes to the configuration of cataract services, which could improve their efficiency and enable them to cope with increasing demand. To address this, a meeting was organised with the services’ clinical and management teams to explore possible alternative actions to achieve this objective. The management team made it very clear from the outset that the actions to be evaluated had to be implementable and that any scenario, which is unrealistic should not be considered. This was driven by the management team decision that the scenario associated with the best performance improvement will be implemented in the real world as the services are under huge pressure and running at full capacity.

Given that cataract services are part of a larger hospital, it was concluded that a radical change to the pathways and the patients’ journey is not possible at the present time. Similarly, as surgery theatres are shared with other services and because it was not expected, at least in the near future, that additional theatres will be built in the hospital, scenarios involving additional “lists” to cataract services were deemed infeasible. This meant that scenarios involving changes to the value of the parameter “number of lists per theatre per week” were excluded from the evaluation.

Following the exclusion of the above cited scenarios, the clinical team indicated that changes to the patients’ management process and use of new technologies can be combined to increase the number of surgeries per list without impacting on care quality or the safety and wellbeing of patients. The management team confirmed that this was feasible and, as a result, a number of scenarios were selected for simulation. As the new processes and technologies are expected to enable more surgeries to take place in every cataract services “list” (half day surgery theatre slot), the scenarios were represented in the model through changes to the value of the parameter “number of patients per list”. Four scenarios were simulated, the first one reflecting the current situation (Baseline Scenario) where the value of the parameter “number of patients per list” is 6. Three alternative scenarios were selected for evaluation and these are as follows:

### Scenario 1

Number of surgeries per list increases from 6 to 7. This is achieved by admitting patients earlier to perform the pre-surgery tests, especially the process of dilating the eye (as this sometimes takes a long time for some patients), and taking the measurements of the lens.

### Scenario 2

Number of surgeries per list increases from 6 to 8. This is achieved by admitting patients earlier to perform the pre-surgery tests and using a new 3D imaging machine to scan the eye and provide a rapid and more accurate measurements of the lens (especially for patients with advanced and hard cataract who sometimes require several rounds of measurements involving both nurses and doctors). The imaging machine uses a rotating camera, which takes images from different angles on the eye. These images are then combined to create a very accurate 3D map of the eye from the anterior chamber to the lens and iris.

### Scenario 3

Number of surgeries per list increases from 6 to 9. This is achieved by admitting patients earlier to perform the pre-surgery tests, using a new 3D imaging machine to provide rapid and more accurate measurements of the lens, and preparing the lens slightly earlier during the surgery so it is ready in time for insertion into the eye.

Each scenario was run for a period of 1 year and the values of the performance indicators to evaluate the different scenarios were collected at the end of the simulation runs.

### Simulation scenarios findings

Following the simulation of the baseline and the three alternative scenarios, results were collected on a set of performance indicators related to the objectives of the research. Although the primarily focus is to determine the service configurations, which could increase the number of cataract surgeries performed, the results analysis was extended to other performance indicators to enable a more comprehensive evaluation (the definition of the performance indicators is presented in Table 1). The analysis of the simulation results is as follows:

**Table 1:** Definition of the performance indicators

Performance Indicator	Definition
Number of surgeries	Total number of surgeries performed in the hospital cataract services per year.
Doctors hours	Total number of hours spent by doctors diagnosing patients, follow-up appointments and cataract surgeries per year
Nurses hours	Total number of hours spent by nurses on all aspect of ophthalmology services including cataract surgeries per year
Consultation room utilisation	Fraction of time (in percentage) of usage of pre-surgery consultation rooms.
Recovery chair utilisation	Fraction of time (in percentage) of usage of post-surgery recovery chair.
Recovery bed utilisation	Fraction of time (in percentage) of usage of post-surgery recovery bed.
Revenue	Total annual amount of money received by the hospital cataract services for performing surgeries and other related activities
Cost	Total annual cost incurred by the hospital cataract services for performing surgeries and other related activities
Surplus	Difference between the total annual revenue and the total annual cost for performing surgeries

#### Number of surgeries

**Fig. 3:**
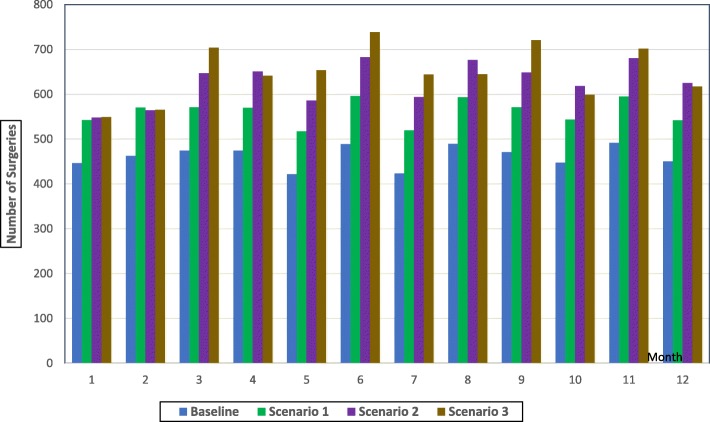
Total number of surgeries per month

**Fig. 4:**
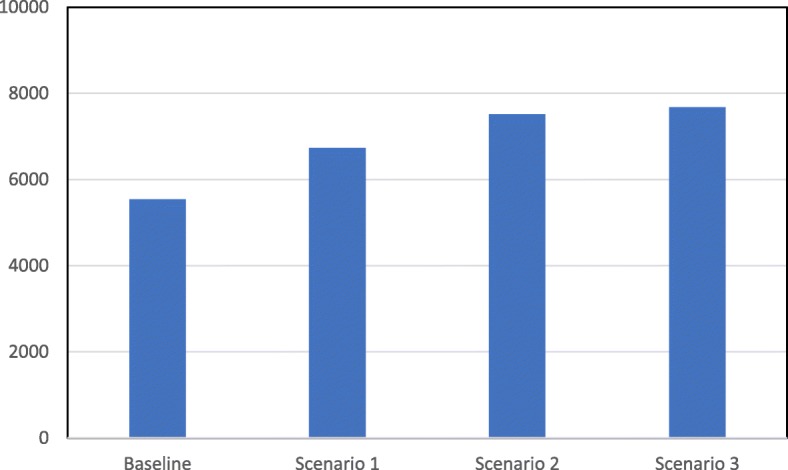
Total number of surgeries per year

The results related to the number of surgeries are presented in Table 2, Fig. 3 (per month) and Fig. 4 (total per year). The results indicate that the total number of surgeries performed per year has an upward trend as we move from the baseline scenario to scenario 3 (Fig. 4). The total number of surgeries per year is expected to move from 5542 under the baseline scenario, to 6731, 7521, and 7682 under scenarios 1, 2, and 3 respectively. This represents a significant improvement and for scenarios 2 and 3, the percentage increase compared to the baseline scenario is 36 and 40% for these respective scenarios.

**Table 2:** Total number of cataract surgeries, doctors hours, and nurses hours (Monthly and Annually)

Month	Number of Surgeries				Doctors Hours				Nurses Hours			
	Baseline	Scenario 1	Scenario 2	Scenario 3	Baseline	Scenario 1	Scenario 2	Scenario 3	Baseline	Scenario 1	Scenario 2	Scenario 3
1	447	542	548	549	432	475	440	363	381	440	414	349
2	463	571	564	565	486	544	492	463	411	486	459	434
3	474	571	647	704	537	596	597	589	440	516	522	516
4	475	570	651	642	517	576	573	532	432	504	511	476
5	422	517	586	654	496	559	556	560	401	478	485	486
6	489	596	683	738	548	615	616	607	454	537	540	534
7	423	519	594	644	484	551	549	541	398	472	477	468
8	489	593	677	645	525	580	582	525	439	512	522	484
9	471	571	648	721	541	609	607	610	442	523	530	528
10	448	544	618	599	478	531	529	484	405	473	473	443
11	492	595	680	702	545	611	613	583	453	533	536	519
12	450	542	625	618	494	548	548	511	412	482	487	454
Total	5542	6731	7521	7682	6083	6795	6703	6369	5067	5955	5958	5690

However, it is noticeable that the relationship between the total number of surgeries per year and the scenarios is non-linear. As we move from the baseline scenario to scenario 3, the number of surgeries per list increases by one surgery from one scenario to the next. However, the rate of improvement in the total number of surgeries per year follows a decreasing slope. The percentage increase is 22% from baseline to scenario 1 (5542 to 6731), 12% from scenario 1 to scenario 2 (6731 to 7521), and a mere 2% from scenario 2 to scenario 3 (7521 to 7682). This counterintuitive finding has two causes. First, some patients scheduled for surgery are found not fit for it during the pre-surgery assessment check (because of high blood pressure for example) causing a cancellation of the surgery. Second, many patients, especially the elderly (the population group mostly affected by cataract), miss their surgery appointment (as a result of illness for example). Consequently, the higher the number of surgeries per list, the higher is the number of cancelled surgeries. As these are elective surgeries planned in advance, once a patient’s surgery is cancelled, the surgery slot for that patient is “lost” as it cannot be allocated to another one. This causes a decrease in the total number of surgeries taking place per list and the cumulative effect of this over time leads to the non-linear relationship observed in the results (in fact, Fig. 3 shows that, in some months, the expected total number of surgeries per month under scenario 3 is lower than that under scenario 2).

#### Utilisation of doctors and nurses

**Fig. 5:**
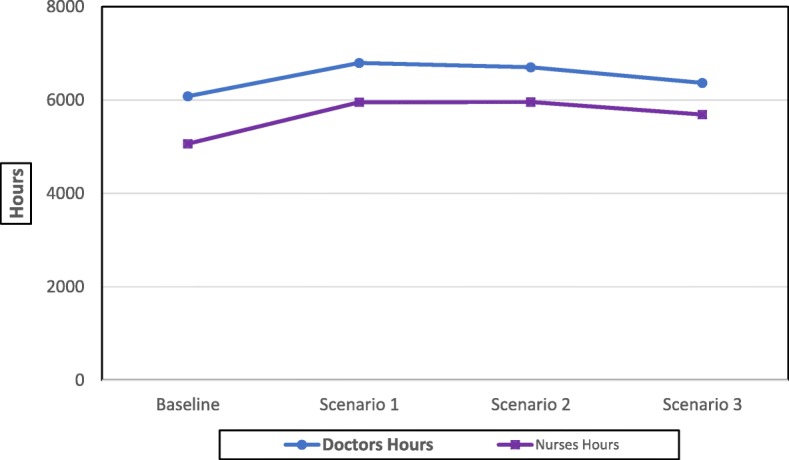
Total number of hours of doctors and nurses per year

Utilisation, in this context, is measured by the total number of hours per year spent by doctors and nurses performing all surgery related activities, which include the pre-assessment tests and the surgery itself (see Table 2 and Fig. 5). The trend in the total number of hours per year is similar for doctors and nurses. It increases from baseline to scenario 1 and, decreases slightly from scenario 1 to scenario 2 and then more importantly from scenario 2 to scenario 3. For doctors, hours increase by 12% from baseline to scenario 1, decrease by around 1% from scenario 1 to scenario 2, and by 5% from scenario 2 to scenario 3. Regarding nurses, hours increase by 18% from baseline to scenario 1, remain virtually constant from scenario 1 to scenario 2, and decrease by 5% from scenario 2 to scenario 3. This trend is due to the fact that the changes to processes and technologies introduced under the alternative scenarios will reduce the time required to complete the surgery related activities for patients. As the total number of surgeries per year under scenario 1 is 22% higher than baseline (see Fig. 4), the same trend occurs in the number of doctors and nurses hours, but with a reduced rate of increase (12 and 18% for doctors and nurses, respectively) due to the fact that the time for a single surgery decreases from baseline to scenario 1 (as it is more likely the eye is properly dilated and the measurements of the lens are confirmed as the surgery starts). The total number of doctors’ hours and nurses' hours are almost similar under scenarios 1 and 2 as the increase in the total number of surgeries between these two scenarios (12% more under scenario 2) is offset by the reduction in the duration of the surgery related activities for a patient (especially eye scanning and measurements of the lens). As the number of surgeries under scenario 3 is higher by only 2% compared to scenario 2, the impact of the surgery duration reduction under scenario 3 (due to the readiness of the lens) is more significant causing a reduction of 5% in the total doctors and nurses hours.

The scale of productivity improvement (due to the reduction in single surgery duration) is much clearer if we compare scenario 3 with the baseline scenario. Although the total number of surgeries per year is 40% higher (from 5542 to 7682) under scenario 3 than under the baseline one, the increase in the total number of hours per year, from the baseline scenario to scenario 3, is only 5% (from 6083 to 6369) for doctors and 12% (from 5067 to 5690) for nurses.

#### Utilisation of resources

**Fig. 6:**
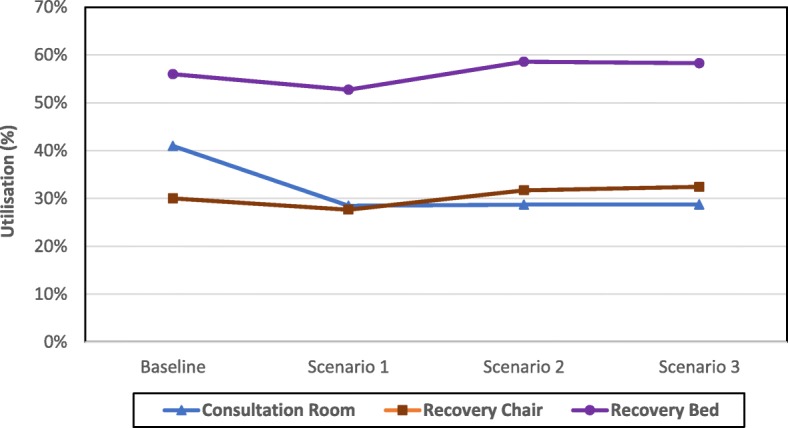
Average utilisation of non-human resources

These are the non-human resources required for surgery activities, namely pre-surgery rooms, post-recovery chairs, and post-recovery beds (surgery theatres are not included in the analysis as they are used at 100% under all scenarios). Results regarding the average utilisation of these resources are presented in Table 3 (monthly and annually) and Fig. 6 (Annually). It is clear that the adoption of any of the alternative scenarios will lead to a marked decrease in the utilisation of the pre-surgery room. The utilisation is reduced by about a third from 41 to 28%, 29, and 29% under scenarios 1, 2, and 3 respectively. However, the trend is slightly different at the post-surgery phase as the average utilisation of recovery chairs and recovery beds decreases when moving from baseline to scenario 1 and then reverse to an ascending trend under scenarios 2 and 3. A change from baseline to scenario 1 should lead to a decrease of 8 and 6% utilisation in recovery chairs and recovery beds respectively whereas a change from scenario 1 to scenarios 2 and 3 is expected to lead to an average utilisation increase of 15 and 12% for recovery chairs and recovery beds respectively. However, if we compare the baseline scenario to scenario 3, the increase of the average utilisation is 8 and 4% for recovery chairs and recovery beds respectively.

**Table 3:** Average percentage utilisation of non-human resources (Monthly and Annually}

Month	Pre Surgery Room Utilisation				Recovery Chair Utilisation				Recovery Bed Utilisation			
	Baseline	Scenario 1	Scenario 2	Scenario 3	Baseline	Scenario 1	Scenario 2	Scenario 3	Baseline	Scenario 1	Scenario 2	Scenario 3
1	29%	21%	21%	21%	29%	28%	28%	21%	57%	54%	57%	47%
2	36%	25%	26%	26%	30%	28%	27%	27%	56%	53%	53%	52%
3	45%	31%	31%	31%	30%	28%	32%	35%	53%	52%	58%	60%
4	40%	28%	29%	29%	31%	28%	33%	32%	57%	52%	60%	58%
5	47%	33%	33%	33%	30%	27%	33%	38%	56%	54%	61%	64%
6	43%	30%	30%	30%	30%	28%	32%	36%	56%	53%	57%	63%
7	45%	31%	31%	31%	30%	28%	33%	37%	56%	53%	60%	63%
8	38%	26%	26%	27%	30%	28%	33%	30%	57%	55%	60%	57%
9	46%	32%	32%	32%	30%	28%	32%	37%	55%	52%	59%	62%
10	38%	26%	26%	27%	30%	27%	33%	30%	56%	52%	59%	55%
11	42%	30%	29%	30%	30%	28%	33%	34%	56%	52%	59%	60%
12	40%	28%	28%	28%	30%	27%	33%	32%	57%	49%	59%	58%
Average	41%	28%	29%	29%	30%	28%	32%	32%	56%	53%	59%	58%

Linking these results to the total number of surgeries per year yields a positive outlook in terms of the utilisation of non-human resources. The total number of surgeries per year under scenario 3 is 40% higher compared to the baseline scenario. However, comparing the average resources utilisation between these two scenarios indicate a decrease of a third in pre-surgery room utilisation, an increase of 8% in post-surgery recovery chair utilisation, and an increase of 4% in post-surgery recovery bed utilisation. This means that it is possible to achieve significant efficiency gains under the alternative scenarios.

An analysis of post-surgery resources utilisation shows that recovery beds are used higher than recovery chairs and this observation is valid for all the scenarios including the baseline one. This is due to the fact that cataract affects mainly elderly people who tend to require a recovery bed after surgery (including some who had local anaesthetic). Additionally, the recovery time for these patients is higher than those recovering on a chair following surgery (mainly mid-aged patients who tend to have a better general health than elderly patients).

#### Revenue, cost, and surplus

**Fig. 7:**
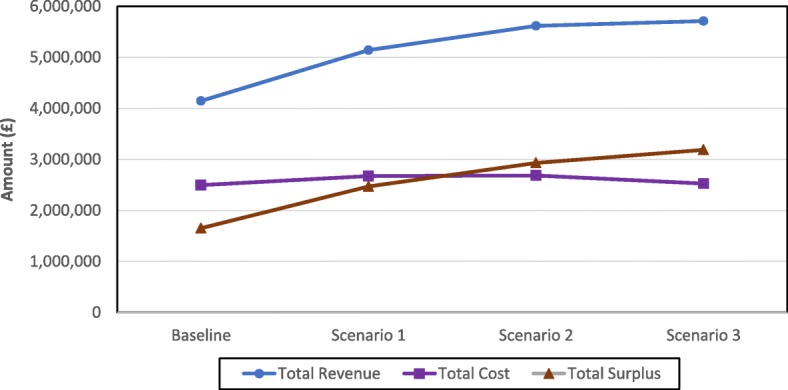
Total revenue, cost, and surplus per year

Results regarding the financial indicators are presented in Table 4 (monthly and annually) and Fig. 7 (annually). It is clear that implementing the alternative scenarios will have a positive impact on the cataract services financial performance. Total revenue per year is expected to increase by about 40% from £4,145,385 under the baseline scenario to £5,712,595 under scenario 3 in line with the increase in the total number of surgeries per year.

**Table 4:** Total revenue, cost, and surplus of the cataract services (Monthly and Annually)

Month	Revenue				Cost				Surplus			
	Baseline	Scenario 1	Scenario 2	Scenario 3	Baseline	Scenario 1	Scenario 2	Scenario 3	Baseline	Scenario 1	Scenario 2	Scenario 3
1	314,075	389,335	392,785	335,035	54,645	63,414	61,306	50,764	259,430	325,921	331,479	284,271
2	339,845	425,330	421,580	422,630	58,979	69,358	65,842	63,914	280,866	355,972	355,738	358,716
3	361,215	444,520	490,270	524,695	62,688	72,617	76,971	79,566	298,527	371,903	413,299	445,129
4	352,375	435,485	483,410	478,010	61,525	71,329	75,656	72,333	290,850	364,156	407,754	405,677
5	327,355	408,985	450,835	491,185	56,866	66,922	70,750	74,695	270,489	342,063	380,085	416,490
6	368,445	459,080	510,905	544,430	64,294	75,342	80,346	82,734	304,151	383,738	430,559	461,696
7	324,255	405,610	450,385	480,010	56,285	66,501	70,628	72,786	267,970	339,109	379,757	407,224
8	361,375	445,845	496,245	477,270	62,890	72,963	77,846	72,479	298,485	372,882	418,399	404,791
9	360,270	447,520	494,995	538,720	62,687	73,264	77,737	81,818	297,583	374,256	417,258	456,902
10	330,180	408,690	453,165	441,165	57,579	66,928	70,968	66,953	272,601	341,762	382,197	374,212
11	370,290	457,880	509,030	521,705	64,421	75,054	79,942	79,179	305,869	382,826	429,088	442,526
12	335,705	412,890	462,465	457,740	58,648	67,928	72,577	69,572	277,057	344,962	389,888	388,168
Total	4,145,385	5,141,170	5,616,070	5,712,595	2,494,787	2,672,338	2,685,529	2,525,564	1,650,598	2,468,832	2,930,541	3,187,031

The trend of the total cost per year has the same shape as that of the total number of hours for doctors and nurses. They increase by around 7% from the baseline scenario to scenario 1, remain slightly flat from scenario 1 to scenario 2, and then decrease by 6% from scenario 2 to scenario 3. As a result, the total cost per year under scenario 3 (£2,525,564) is a mere 1% higher than that of the baseline scenario (£2,494,767).

The total surplus per year, which is the difference between the total revenue per year and total cost per year, follows an ascending trend reaching a maximum percentage increase of 93% from £1,650,598 under the baseline scenario to £3,187,031 under scenario 3. This is due to the fact that the total cost per year is virtually the same under these two scenarios whereas the total revenue per year under scenario 3 is considerably higher in comparison to the baseline scenario (total revenue is driven by the total number of surgeries which is higher under scenario 3). It is, therefore, possible to conclude that the changes suggested under the alternative scenarios will enable the cataract services to achieve a robust financial performance as well as considerable efficiency gains.

## Discussion

The results of this study provide evidence that coping with the high demand for cataract surgery is possible through introduction of new processes and medical technologies. This finding is important as demand for cataract surgery is already high and expected to increase sharply in the near future. As significant investment in additional surgery theatres capacity is a distant prospect due to the NHS funding constraints, it is important to find other ways to deal with demand. Implementing innovative processes and technologies to reduce time and waste performing surgery related activities, a core principle of lean operations in healthcare [47], offer promising possibilities to NHS managers to adapt to the new “normal” of “doing more with less”.

The expected benefits from introducing the new processes and treatment technologies are numerous. From a cost perspective, the increased number of surgeries is virtually cost neutral as results show that the total cost per year will be virtually the same. The expected surplus will cover any investment in new equipment, which is important for an organisation suffering from budget restrictions like the NHS. Furthermore, the innovations are expected to lead to better quality of care, enhanced safety of the surgery, and improved clinical results for patients.

The other positive outcome from the interventions relate to the utilisation of resources (human and non-human). Productivity gains are expected to materialise as the rate of increase in the total number of surgeries markedly exceeds that of doctors and nurses hours. Similarly, non-human resources utilisation results are positive as pre-surgery room utilisation goes down by a third and post-surgery recovery chair and post-surgery recovery bed utilisations increase marginally.

The expected gains from redesigning the cataract services cannot, however, disguise the existence of issues, which warrant special attention from the services’ management and clinical teams. The expected increase in cancelled surgeries under the alternative scenarios needs to be addressed. For example, pre-surgery checks can be carried out very close to the date of surgery so that patients found to have health issues, which prevents surgery can be rescheduled to a later date. Non-attendance of surgeries (especially by the elderly) can be reduced through proactive communication with patients (or families and carers where relevant) to make sure they are planning to attend the surgery. Reminders through phone calls and mobile phone messaging have been found to reduce “no shows” of patients [48, 49]. Another possible lean operations policy is to schedule surgeries for patients with high risk of no-attendance in the same surgery list and overbook the list as this was found to reduce the number of cancelled surgeries [50].

Utilisation of non-human resources is another area of concern. There is a clear imbalance along the surgery pathway as theatres are used at full capacity whereas pre-surgery rooms and post-surgery recovery beds and chairs are under-utilised (under scenario 3, utilisation of pre-surgery rooms and post-surgery recovery chair is 29 and 32% respectively). Cataract services and hospital managers should review this situation by, for example, converting some of the non-used capacity into surgery theatres or use it to provide care to patients suffering from other eye conditions (eg Glaucoma).

The clinical and management teams need to ensure that the efficiency and productivity improvements are sustainable in the future. In many situations, short term success following implementation of lean operations policies is not accompanied by the necessary commitment from top management to identify and adapt to new challenges. This was cited as one of the main reasons for the failure of lean operations in healthcare [19].

The DES model provides managers with evidence for re-designing the cataract services and finding the most efficient way to deliver care to patients. In addition, the friendliness and ease of use of DES software enabled them to be fully engaged with the development of the model and the implementation of the recommended changes [51].

This research has some limitations and can be extended in a number of ways. The model could be used in other hospital settings to determine if the expected improvement can also be achieved elsewhere. The pathways can be extended to include more detailed representation of out-patient and community services. Patients’ satisfaction performance indicators could be added to the operational and cost indicators analysed in the current study. Finally, policies to reduce non-attendance of surgery appointments can be added to the model to evaluate their impact. This additional research will shed more light on cataract services and provides a strong evidence regarding the best policies to implement to cope with future demand, achieve sustainable efficiency improvement, and enhance patients’ quality of care and satisfaction.

## Conclusions

The incidence and prevalence of cataract is high and raising in the UK as a consequence of the ageing population. As surgery is the only possible medical option to treat it, the number of surgeries expected to take place in coming years is phenomenal. Given that this is taking place against the background of sustained austerity and constrained public finances, findings new ways to achieve “more with less” is a top priority for cataract services decision makers in the UK.

Motivated by the scale of the challenge, we developed a DES computer model to aid key decision makers in establishing whether possible changes, based on the principles of lean operations, could improve the operational and financial performance of cataract services. The model was used to test possible alternative scenarios involving the use of new processes and technologies and the results indicated a very positive outcome on various performance indicators.

The study identified non-attendance of surgeries as a serious issue for cataract services. This means that proactive management of and communication with patients is as important as the changes to processes and technologies. This finding is critical as lean operations tend to focus mainly on the process side of healthcare and does not adequately deal with the patients’ behavioural aspects.

The positive outcomes associated with the policies evaluated in this study may hide a threat regarding the sustainability of lean operations application to cataract services. As there is evidence that short term gains sometimes led to complacency, it is important that top decision makers need to be fully committed to and supportive of the lean operations principles to avoid the failures described in the literature.

To conclude, this study showed that innovative ways of designing patients’ treatment and care pathways can lead to substantial improvements in contexts facing significant challenges such as health services. This is welcomed as the ability to deliver timely and high quality care to expanding populations is critical to ensure the well-being of individuals and the prosperity of societies.

## Additional file

Additional file 1: List and values of the parameters in the DES model. The data include the list of the parameters included in the DES model and the numerical values of these parameters. The data represents the situation of the cataract services where the model development was conducted. The data parameters values were entered in the DES model so that it can be run to simulate the current state of cataract services. This simulation run generated the “baseline” scenario results for the model. (DOCX 14 kb)

## Abbreviations

DES: Discrete Event Simulation
DoH: Department of Health
NHS: National Health Service
UK: United Kingdom
