# Supplementary material for: A discrete event simulation model to evaluate the treatment pathways of patients with cataract in the United Kingdom
Source: BMC Health Serv Res. 2018 Dec 4;18:933. doi: 10.1186/s12913-018-3741-2 (PMC6278024; doi:10.1186/s12913-018-3741-2)
Supplement: Supplementary file 1 — List and values of the parameters in the DES model. The data include the list of the parameters included in the DES model and the numerical values of these parameters. The data represents the situation of the cataract services where the model development was conducted. The data parameters values were entered in the DES model so that it can be run to simulate the current state of cataract services. This simulation run generated the “baseline” scenario results for the model. (DOCX 14 kb) [file 12913_2018_3741_MOESM1_ESM.docx]

| **Parameter** | **Description** |
| --- | --- |
| Total number of patients with cataract per year | 6,295 (Forecasted on a monthly basis using HES dataset) |
| Percentage of patients referred from an accredited optometrist, non-accredited , and other services in the hospital. | Accredited optometrist = 18.5%  Non-accredited optometrist = 74.3%  Other services = 17.2% |
| Percentage of patients having cataract surgery as day case, outpatient attendance, and inpatient (staying overnight in hospital) | Inpatient Admission = 0.37%  Day case = 98.49%  Outpatient attendance = 1.14% |
| Percentage of patients not suitable for surgery | 5% |
| Duration of an eye examination (in mins) | 15 mins |
| The time required to anaesthetize a patient (in mins) | 15 mins |
| The duration of surgery (in mins) | 20 mins |
| Waiting time for the first appointment (in days) | 40 days |
| Waiting time for pre-operative assessment (in days) | Average=10 days. Distribution generated from HES data and entered into model’ |
| Waiting time for surgery (in days) | Average=133 days. Distribution generated from HES data and entered into model’ |
| Number of surgery theatres | 3 |
| Number of lists per theatre per week | 6 |
| Number of patients per list | 6 |
| Number of outpatient consultation rooms | 2 |
| Number of pre-surgery assessment rooms | 2 |
| Number of anaesthetic rooms | 2 |
| Number of recovery beds | 3 |
| Number of recovery chairs | 4 |
| Number of surgeons | 6 |
| Number of nurses | 3 |
| Number of theatre specialists | 3 |
| Number of anaesthetists | 1 |
| Number of healthcare assistants | 3 |
| Each staff availability in percentages | Nurse = 100%  Consultants (surgery) = 100%  Consultants ( follow-up review) = 100%  Consultant (outpatient examination) = 30% |
| National tariff for cataract surgery: inpatient admissions | £900 |
| National tariff for cataract surgery: day case | £600 |
| National tariff for cataract surgery: outpatient | £600 |
| National tariff for first outpatient appointment | £140 |
| National tariff for follow-up appointments | £85 |
| Hourly cost of a doctor | £48.64 |
| Hourly cost of a theatre specialist | N/A |
| Hourly cost of a nurse | £22.62 |
| Hourly cost of an anaesthetist | £33.23 |
| Hourly cost of a healthcare assistant | £17.66 |
| Other costing per patient (e.g. estate costs) | Cost of lens = £50 |
